# Case series: Transient neonatal macular retinoschisis - a developmental phenomenon in term infants

**DOI:** 10.1038/s41433-025-04082-9

**Published:** 2025-10-23

**Authors:** Ravi Purohit, Helena Lee, Lewis Smith, Rachel Edminson, Frank Proudlock, Irene Gottlob, Kanmin Xue, Chetan K. Patel

**Affiliations:** 1 Oxford Eye Hospital, Oxford University Hospitals NHS Foundation Trust, Oxford, UK; 2 University of Leicester Ulverscroft Eye Unit, Robert Kilpatrick Clinical Sciences Building, Leicester Royal Infirmary, Leicester, UK; 3 Clinical and Experimental Sciences, Faculty of Medicine, University of Southampton, Sir Henry Wellcome Laboratories, Southampton University Hospital, Southampton, UK; 4 Eye Unit, University Hospital Southampton NHS Foundation Trust, Southampton, UK; 5 Nuffield Laboratory of Ophthalmology, Nuffield Department of Clinical Neurosciences, University of Oxford, Oxford, UK; 6 Great Ormond Street Hospital for Children NHS Foundation Trust, London, UK

**Keywords:** Retina, Retina

## Abstract

**Aims/Purpose:**

To report and characterise a novel observation of ‘transient neonatal macular retinoschisis’ (TNMR) in a series of healthy, term infants, differentiate it from congenital pathologies, such as X-linked retinoschisis (XLRS).

**Methods:**

This case series reports on five term neonates with incidental macular retinoschisis identified during a prospective developmental study or routine ophthalmic assessment. High-resolution spectral-domain optical coherence tomography (OCT) was used for initial imaging and for longitudinal follow-up over several months to monitor the findings.

**Results:**

All five infants presented with macular retinoschisis, most commonly between the inner nuclear and inner plexiform layers. TNMR resolved spontaneously in all cases, with foveal anatomy appearing normal on follow-up imaging between 44 and 94 weeks post-menstrual age. In one infant with a family history of XLRS, complete resolution and negative *RS1* genetic testing ruled out the inherited condition.

**Discussion:**

TNMR appears to be a self-limiting phenomenon. We explore its mechanistic relevance, proposing it is a benign variant of normal foveal maturation, offering new insight into this process. We consider its relevance to macular oedema in retinopathy of prematurity (ROP), suggesting a developmental continuum with changes in preterm infants. Spontaneous resolution is the key feature distinguishing TNMR from congenital conditions like XLRS. Recognition of this entity is clinically important to avoid misdiagnosis and unnecessary investigations.

## Introduction

Advances in optical coherence tomography (OCT) technology have improved access to high-resolution retinal imaging for young children and neonates [1–3], allowing detailed assessment of early macular structures. Whilst studies describing postnatal development of the fovea suggest that the fovea of newborn infants has a well-formed pit with a thin outer nuclear layer [4], our work has identified a novel phenomenon we term ‘transient neonatal macular retinoschisis’ (TMNR) in otherwise healthy full-term neonates. This phenomenon, characterised by spontaneous resolution within weeks to months of macular layer separation, presents a distinct clinical picture that needs to be differentiated from established conditions such as X-linked retinoschisis (XLRS) which may present with separation of retinal layers from birth which may involve the macula or periphery [5], or from macular changes like cystoid oedema typically reported in premature infants [6, 7]. As part of our foveal development study [4], and neonatal retina and craniofacial services at tertiary referral centres, we have been imaging infants, including newborns, using three methods of spectral domain OCT; Heidelberg Spectralis Flex Module (Heidelberg Engineering, Heidelberg, Germany) [2], hand-held Leica Envisu C2300 (Leica Microsystems GmbH, Wetzlar, Germany) [3], and ‘flying baby’ Heidelberg Spectralis HRA [1]. Herein, we report a series of incidental findings of macular retinoschisis in five neonates born near full term, which resolved spontaneously with age. Our observations would suggest that TNMR may be part of normal vitreo-retinal development in a small proportion of infants. This developmental phenomenon needs to be borne in mind when evaluating newborns with a family history of XLRS and provides novel insight into the process of fovea maturation. Recognising TNMR is therefore important for understanding the spectrum of neonatal foveal maturation.

Current neonatal OCT literature largely focuses on premature infants, describing findings such as ‘cystoid macular oedema’, or infantile presentation of XLRS. However, there is a paucity of detailed reports on transient macular changes in healthy term infants, creating an important observational gap. To address this lack of literature, our report provides the first detailed characterisation of ‘transient neonatal macular retinoschisis’ (TMNR) in term infants, exploring this novel finding and its implications for understanding the spectrum of foveal maturation.

## Case 1

A 10-week-old male (post-menstrual age, PMA = 47 weeks) was referred to The Oxford Craniofacial Unit for metopic craniosynostosis. He was born by normal vaginal delivery at 37 weeks' gestational age (GA), weighing 4196 g. The macula OCT was captured during the standard-of-care protocol for optic nerve imaging using Heidelberg Spectralis-Flex OCT. His craniosynostosis was non-syndromic and did not result in symptoms of intracranial hypertension. Ophthalmic examination was otherwise unremarkable, and there were no additional general medical problems.

**Fig. 1:**
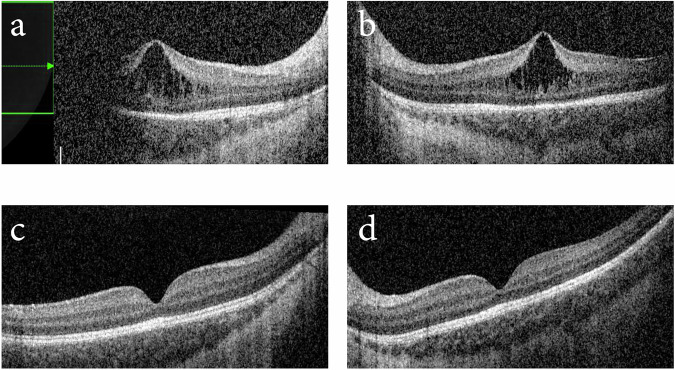
OCT imaging of Case 1. OCT Flex imaging of **a** right fovea and **b** left fovea at 47 weeks PMA showing separation between the INL and IPL, which resolved by 56 weeks PMA in **c** right fovea and **d** left fovea.

The macula OCT revealed a separation between the inner nuclear layer (INL) and inner plexiform layers (IPL) in the foveal region spanning 2046 µm in the left eye. A poor view of the temporal aspect of the right macula did not allow for measurement of the extent of the separation. The maximum retinal elevation appeared to ‘peak’ at the centre of the fovea, where the total retinal thickness was 506 µm in the right eye and 484 µm left (Fig. 1a, b). Repeat OCT imaging was carried out at 19 weeks (PMA = 56 weeks), at which point both fovea resumed normal appearance, with central foveal retinal thickness of 152 and 162 µm for the right and left eye, respectively (Fig. 1c, d).

## Case 2

A 10-week-old male (PMA = 48 weeks) was referred to The Oxford Craniofacial Unit for sagittal craniosynostosis. He was born by normal vaginal delivery at 38 weeks GA and 2820 g. Polyhydramnios and transverse lie were noted in the third trimester. His craniosynostosis was non-syndromic, although paternal Chiari malformation was noted. No other ocular anomaly was noted, and no symptoms of intracranial hypertension were present.

**Fig. 2:**
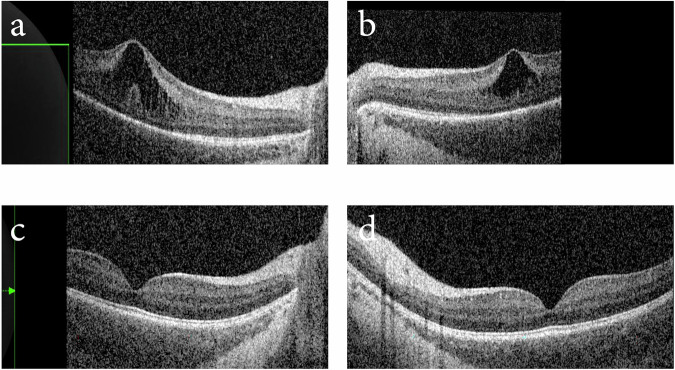
OCT imaging of Case 2. OCT Flex imaging of **a** right fovea showing separation between the ONL and OPL and the INL and IPL and **b** left fovea showing separation between the INL and IPL at 48 weeks. This was resolved by 61 weeks PMA in **c** right fovea and **d** left fovea.

Spectralis-Flex OCT of the macula captured alongside the optic disc revealed asymmetric macular retinoschisis. At the right fovea, schitic separation was present between the INL and IPL spanning 3741 µm, and between the OPL and outer nuclear layer (ONL) spanning 852 µm. The total retinal thickness was 518 µm at the fovea (Fig. 2a). The left eye presented with separation between the INL and IPL spanning 2004 µm with a total retinal thickness of 405 µm at the fovea (Fig. 2b). The macular schisis appeared spontaneously resolved on subsequent OCT imaging at 5 months of life (PMA = 61 weeks).

## Case 3

A 2-week-old male infant was recruited to an ethics-approved study investigating normative postnatal foveal development (national research ethics ref: 12/EM/0261) [4]. He was born at 37 weeks GA, weighing 2608 g, by normal vaginal delivery. He was born with neonatal jaundice and received phototherapy for two weeks.

**Fig. 3:**
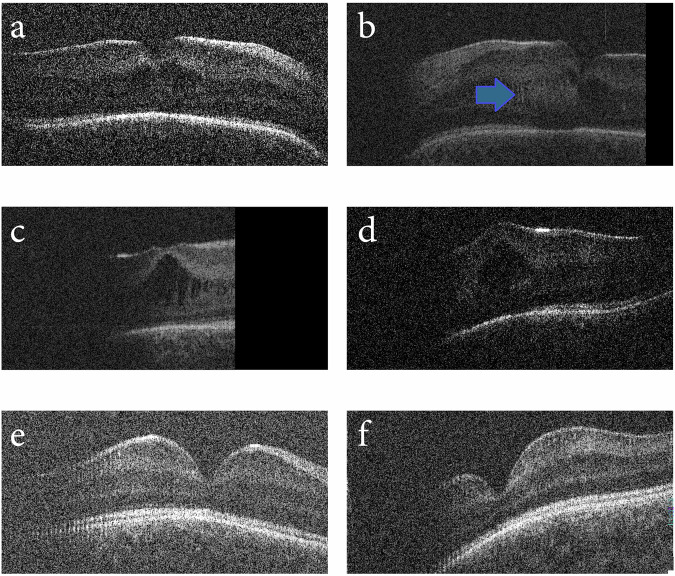
OCT imaging of Case 3. HH-OCT Envisu imaging of **a** right fovea showing separation between the INL and IPL and **b** left fovea showing separation between the INL and IPL, where the arrow shows a hyper-reflective lesion in the nasal macula at 39 weeks PMA. **c** The separation between the INL and IPL increased in the right eye at 42 weeks PMA. **d** resolution of the hyper-reflective lesion was seen in the left eye with greater separation between the ONL and OPL. This resolved by 48 weeks PMA in **e** right fovea and **f** left fovea.

OCT imaging using a hand-held OCT (Leica Envisu C2300) at two weeks after birth (PMA = 39 weeks) revealed a shallow foveal pit and thickened retina with schitic separation between the INL and IPL in both eyes (Fig. 3a, b) a hyper-reflective region within the ONL on the nasal aspect of the left fovea. At five weeks of age (PMA = 42 weeks), the separation between retinal layers had increased bilaterally, however the hyperreflective lesion had regressed (Fig. 3c, d). By 11 weeks of age (PMA = 48 weeks), the schitic changes appeared resolved with both maculae appearing within normal limits (Fig. 3e, f).

## Case 4

**Fig. 4:**
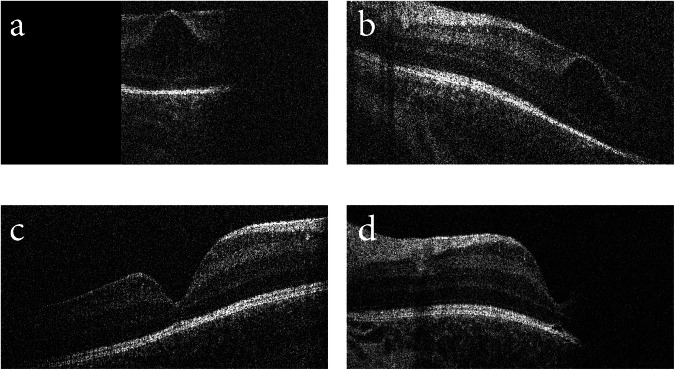
OCT imaging of Case 4. HH-OCT Envisu imaging of **a** right fovea showing separation between the INL and IPL and **b** left fovea showing separation between the INL and IPL at 42 weeks. This was resolved by 44 weeks PMA in the **c** right fovea and **d** left fovea.

A female infant born at 37 weeks GA and 3207 g was also recruited as part of the study of normative postnatal foveal development [4]. She had neonatal jaundice that was treated with phototherapy in the neonatal care unit. Hand-held OCT imaging using Leica Envisu C2300 at two weeks of age (PMA = 39 weeks) revealed inner retinal schitic changes at both fovea with separation between the INL and IPL (Fig. 4a, b). Repeat OCT imaging at 7 weeks of age (PMA 44 weeks) showed full resolution of the schitic changes (Fig. 4c, d).

## Case 5

A 3-week-old male was referred to the Paediatric Retina Service for investigation of X-linked retinoschisis in view of his maternal grandfather being affected by the disease. He was born at 38 weeks GA and 2760 g by normal vaginal delivery. The infant was otherwise medically well.

**Fig. 5:**
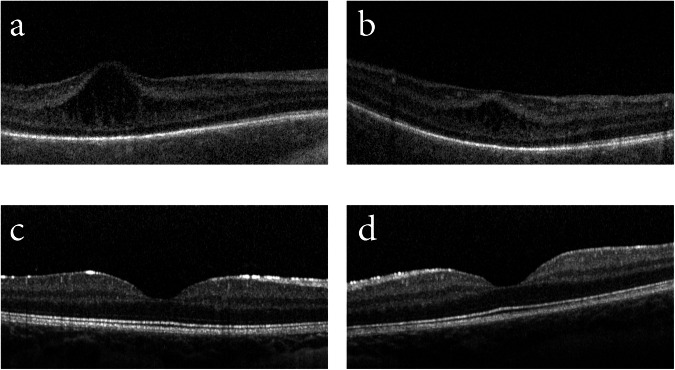
OCT imaging of Case 5; an XLRS suspect. Spectralis OCT of **a** right fovea and **b** left fovea showing separation between the INL and IPL at 42 weeks PMA. This was resolved by 94 weeks PMA in the **c** right fovea and **d** left fovea.

OCT imaging was carried out on a Heidelberg Spectralis-HRA using the flying baby technique [1] at one month of life (PMA = 42 weeks). Schitic separation was observed between the ONL and OPL at both fovea, spanning 1864 µm in the right eye and 1618 µm in the left eye. Total central foveal thickness was 497 µm and 374 µm in the right and left eye, respectively (Fig. 5a, b). Based on these OCT findings, a clinical diagnosis of X-linked retinoschisis was initially made. However, subsequent genetic testing did not identify any mutations in the retinoschisin (*RS1*) gene associated with XLRS. Repeat OCT imaging at 13 months (PMA = 94 weeks) of age using a modified Heidelberg Spectralis-HRA under anaesthesia showed resolution of the macular retinoschisis and normal fovea anatomy in both eyes. Central foveal thicknesses of 202 and 201 µm were measured in the right and left eye, respectively (Fig. 5c, d).

## Discussion

This case series represents the first observations of TNMR in full-term infants whose clinical profiles are distinct from non-premature neonates with severe comorbidities previously described with macular oedema [8]. Although these cases demonstrate the clinical utility of three methods of infant OCT image acquisition, the requirement and availability of retinal OCT imaging for newborns remain uncommon, therefore we suspect the incidence of TNMR may be under-appreciated. Two infants from a cohort of 46 enroled in our retinal development study, who were imaged between 37-52 weeks PMA, were identified with TMNR and subsequently excluded from that study due to this finding [4]. Based on the detection of two cases, we would venture to estimate an incidence of TNMR of around 4% (2/46). Similar foveal changes have previously been described as ‘cystoid macular oedema’ in premature infants with and without retinopathy of prematurity (ROP), with an incidence of 29 to 50% [6, 7]. This term is normally used to describe inflammation-associated intraretinal fluid thought to arise as a leak from macular capillaries. We prefer the term retinoschisis to describe our finding as the pathogenesis is unknown. A weak correlation with ROP stage has been proposed [7, 9], while others have reported no correlation with ROP severity and resolution by 52 weeks PMA [6, 10–12]. Through the ROP ‘lens’, it has been hypothesised that these ‘cystoid changes’ may be the result of elevated levels of vascular endothelial growth factors (VEGF). This would not easily explain their occurrence in premature infants who do not have any ROP. Our observation of TNMR among full-term infants may represent the tail end of the same developmental phenomenon occurring during the third trimester until shortly after birth. It is important to contextualise these pre-term findings. Due to intensive ROP screening, the pre-term population often serves as a ‘de facto’ model for observing early OCT changes, providing a valuable comparative baseline. However, the high prevalence of ‘CMO’ reported in pre-term infants likely reflects this high screening rate. We postulate that similar transient phenomena, termed TNMR, occur in term infants but are likely underreported due to the absence of routine OCT screening. This highlights the need for more studies specifically investigating the term population.

Previous reports describe macular oedema in non-premature infants, often those with significant underlying health conditions, including low birthweight and neurological pathology [8]. Our study, in contrast, characterises TNMR in term infants, some with less severe clinical profiles such as non-syndromic craniosynostosis without raised intracranial pressure or managed jaundice. Our morphological characterisation of these transient changes complements earlier studies focused on functional outcomes of macular oedema. We interpret TNMR as a potential transient developmental variation, distinct from presumed pathological oedema in such medically complex cohorts. We further postulate that the previously reported oedema in comorbid infants and the TMNR we describe may represent related entities on a common developmental spectrum, with clinical manifestations influenced by systemic health and the timing of OCT imaging.

Cases 1 and 2 were both identified in our craniofacial clinic, where patients had metopic or sagittal non-syndromic craniosynostosis, within a timeframe where approximately 100 infants under three months of age underwent OCT imaging. Whilst infants with craniosynostosis are at increased risk for certain ophthalmic conditions such as refractive errors, strabismus, and papilledema secondary to raised intracranial pressure [13–15], intrinsic macular pathology is not a typically reported feature associated with craniosynostosis.

Cases 3 and 4 both had a history of neonatal jaundice. Whilst severe, untreated hyperbilirubinaemia could have significant ophthalmic implications, neonatal jaundice managed with phototherapy is typically associated with favourable ocular outcomes. No significant differences in ocular findings, including retinal appearance, in treated infants compared to controls have been reported [16]. The cases of TMNR among the jaundiced infants were identified from a larger cohort but excluded from the normative developmental study precisely because this finding was considered distinct from the typical developmental trajectory observed in their peers and unlikely to represent a consequence of jaundice. We propose that TMNR may instead signify an alternative developmental trajectory, rendering it a noteworthy phenomenon potentially reflecting an incidental observation. We do not believe there to be a link between craniosynostosis or neonatal jaundice and TNMR; however, these possibilities cannot be definitively excluded based on the small sample size.

Case 5 was unique in having a maternal carrier of XLRS thus a 50% chance of being affected by the condition. The observation of spontaneous resolution of foveal retinoschisis and absence of mutations in *RS1* would make the diagnosis of XLRS very unlikely in this individual. XLRS is a congenital retinal condition that, in some severe instances, has been identified as early as one month of age and can present with complications such as retinal haemorrhage [17]. More commonly, XLRS is diagnosed later in early childhood, often when visual signs like strabismus prompt further investigation [18]. The observation of complete spontaneous resolution in this case would not be in keeping with a typical XLRS diagnosis but highlights that the age of presentation of XLRS and TNMR can overlap, and that caution must be exercised when making early diagnoses of XLRS based on OCT imaging alone.

Retinoschisin is secreted primarily by photoreceptors and localises to the photoreceptor inner segments, the IPL and OPL, which flank the bipolar cells (constituting the INL) in the adult human retina [19]. The protein plays an important role in intercellular adhesion and stabilisation of synaptic junctions between retinal layers. A murine model shows *Rs1* mRNA and retinoschisin proteins to be concentrated at the level of the photoreceptors at birth, before migrating towards the inner retina during postnatal development [20]. It is unclear how the level of *RS1* expression may fluctuate during human retina development or during infancy, when cones are still differentiating, and whether these fluctuations might contribute to transient retinoschisis. In addition, the potential effects of vitreous traction over the macula during regression of primary vitreous may also contribute to the phenomenon seen. Our consistent observation of schitic separation between the INL and IPL would indicate disruption of synaptic connections between bipolar cells and ganglion cells. To a lesser extent, we also observed schitic separation between the INL and OPL, which would suggest some disruption of synaptic connections between bipolar cells and photoreceptors.

The finding of retinoschisis at birth can be very worrying to parents and clinicians. However, this case series demonstrates spontaneous resolution of the schitic changes in all cases, by a PMA of 44 to 56 weeks. The spontaneous resolution to normal foveal anatomy suggests that TNMR is most likely a variant of normal foveal development. However, follow-up of these cases to assess long-term visual function and myopia in early childhood [12] would be warranted; however, if TNMR is a variant of macular oedema seen in ROP, myopia is contested by other reports [9]. The finding of foveal retinoschisis may lead to a false diagnosis of pathology or a prediction of poor visual prognosis. Whilst the small size and short follow-up interval of our cohort preclude definitive recommendations on the precise timing for follow-up, our findings support repeat OCT imaging after several months to monitor for the expected spontaneous resolution of TMNR. It is important to note that although all cases were resolved in our series, this study does not provide direct data to guide management in cases of non-resolution. However, should TNMR persist significantly beyond 56 weeks PMA, or if other atypical clinical features emerge, we recommend a comprehensive clinical assessment, including genetic testing for conditions like XLRS.

Future electrodiagnostic assessment of neonates presenting with TNMR may be able to better correlate the anatomical changes seen with the emergence of retinal function during early infancy. Larger studies with imaging at shorter intervals could more accurately estimate prevalence and further elucidate macular development and aid in differentiating TNMR from true retinoschisis or macular oedema.

## Summary

### What was known before:

- Retinoschisis, characterised by a splitting of the retinal layers, can lead to significant visual impairment.
- While cystoid macular oedema (CMO) has been documented as a complication in preterm infants, its occurrence in term infants has not been reported.

### What this study adds:

- This research reports a physiological separation of retinal layers in full-term infants, characterised by spontaneous resolution.
- The findings presented herein propose a mechanism involving retinoschisin, offering a potential explanation for this phenomenon observed in both term and preterm infants.

## Data Availability

The retinal images that support the findings of this study are not publicly available due to patient confidentiality regulations. The data are available from the corresponding author upon reasonable request.
